# Beyond mood screening: a pilot study of emotional, cognitive, and somatic concerns in patients with Long COVID

**DOI:** 10.3389/fpsyg.2025.1517299

**Published:** 2025-06-17

**Authors:** Tali R. Sorets, John-Christopher A. Finley, W. Curt LaFrance, Ryan Van Patten, Kristen Mordecai, Millenia Jimenez, Stephen Suchy, Joshua Cahan, Igor J. Koralnik, Leora R. Cherney, Erica Cotton

**Affiliations:** ^1^Northwestern University Feinberg School of Medicine, Chicago, IL, United States; ^2^Department of Psychiatry and Behavioral Sciences, Northwestern University Feinberg School of Medicine, Chicago, IL, United States; ^3^Departments of Psychiatry and Neurology, Rhode Island Hospital, Brown University, Providence, RI, United States; ^4^VA Providence Health Care System, Providence, RI, United States; ^5^Veterans Affairs Rehabilitation Research and Development Center for Neurorestoration and Neurotechnology, Providence, RI, United States; ^6^Warren Alpert Medical School of Brown University, Providence, RI, United States; ^7^Baltimore Veterans Affairs Medical Center, Baltimore, MD, United States; ^8^Ken and Ruth Davee Department of Neurology, Northwestern University Feinberg School of Medicine, Chicago, IL, United States; ^9^Shirley Ryan Ability Lab, Chicago, IL, United States; ^10^Department of Physical Medicine and Rehabilitation, Northwestern University Feinberg School of Medicine, Chicago, IL, United States

**Keywords:** Long COVID, SARS-CoV-2 infection, personality assessment inventory, psychopathology, mental health risk factors

## Abstract

**Objective:**

This descriptive pilot study reported the emotional, cognitive, and somatic concerns of a clinically referred sample of patients with Long COVID using a comprehensive psychological measure. These subjective concerns were considered in the context of other psychological characteristics and historical mental health factors.

**Methods:**

The study sample comprised 26 adults with Long COVID who were referred from a neurology COVID-19 clinic for neuropsychological screening based on the patient's cognitive concerns. Empirically established cutoffs from the Personality Assessment Inventory were used to assess clinically elevated emotional, cognitive, and somatic concerns. Preexisting mental health data were obtained via medical records and clinical interview.

**Results:**

Approximately 62 and 50% of the sample had elevated somatic and cognitive concerns, respectively. Additionally, 42% of the sample exhibited elevated emotional concerns associated with depression, but this was primarily driven by the physiological aspects of depression. Between 15–27% of patients had elevated anxiety-related concerns. Over 80% of the sample had previously received psychotherapy and had been diagnosed with a mental health disorder prior to their SARS-CoV-2 infection. Furthermore, over half of the sample reported a history of abuse, and 12–15% had previously attempted suicide or had been hospitalized for psychiatric reasons.

**Conclusions:**

Findings suggest that patients with Long COVID may present with extensive psychiatric histories and various somatic, cognitive, and emotional concerns. These psychological characteristics may be important for the treatment of Long COVID but may be overlooked using screening measures.

## Introduction

Based on self-report data, one in four adults with COVID-19 may not follow a typical recovery trajectory, reporting persistent symptoms for >12 weeks post-infection (CDC, 2023). This condition is termed Post-Acute Sequelae of SARS-CoV-2 Infection, or colloquially referred to as “Long COVID” (Soriano et al., 2022). Long COVID may not only manifest as somatic concerns (e.g., fatigue or shortness of breath), but also as cognitive (e.g., attentional lapses) or emotional concerns (Perez Giraldo et al., 2023; Cabrera Martimbianco et al., 2021; Cahan et al., 2024; Schretlen et al., 2024; Taquet et al., 2021). There is a wealth of literature examining all these subjective concerns in Long COVID. However, most of these studies have relied on brief screening instruments, have not examined multiple aspects of somatic, emotional, and cognitive concerns in individuals with Long COVID, and have not included a thorough description of historical mental health factors that may influence these concerns (for review, see: Alkodaymi et al., 2022; Montani et al., 2022; Nalbandian et al., 2021; Oronsky et al., 2023; Marchi et al., 2023). Further investigation is necessary, as such concerns and historical factors can impair quality of life and impact treatment outcomes (Lemogne et al., 2023; Carfì et al., 2020).

Emotional concerns including anxiety, depression, and acute stress are commonly described in patients with Long COVID (Méndez et al., 2021; Taquet et al., 2021). However, the relationship between COVID-19 and these concerns remains complicated by the fact that most studies assess anxiety and depression via narrowly focused self-report screening instruments (Bourmistrova et al., 2022; Montani et al., 2022). Depression and anxiety are just two of the many potential emotional concerns following COVID-19 but are also heterogeneous constructs that can manifest as physiological, affective, or mental distress (Thase, 2013; Kung et al., 2021; Szuhany and Simon, 2022). Identifying other aspects of emotional distress (e.g., stress, mania, obsessive-compulsions) and differentiating them based on how they are experienced by the individual may help identify treatment targets (Serafini et al., 2023; Renna et al., 2017**)**. However, screening measures are not typically designed to distinguish between various forms or presentations of psychopathology (Newson et al., 2020; Kotov et al., 2017). Another limitation of these measures is that they generally assess only current symptoms. It may be important to determine whether the emotional concerns identified via screening measures reflect merely the individual's current state or represent more enduring personality traits (Engelmann et al., 2024**)**; this is based on recent studies showing that personality traits, such as neuroticism, may affect how individuals experience somatic symptoms (e.g., fatigue) in Long COVID (Delgado-Alonso et al., 2022).

Consistent with research on other persistent syndromes following acute infection or injury (van Gils et al., 2020; Galvez-Sánchez et al., 2018), there are likely interdependent associations between somatic, cognitive, and emotional concerns in patients with Long COVID (Engelmann et al., 2024). For these reasons, researchers should focus not only on emotional concerns but also on how these may relate to subjective somatic and cognitive issues in individuals with Long COVID. However, most existing research has been unsuccessful in capturing these interdependencies, relying on screening measures that assess only one type of concern (Zimmerman, 2024). Furthermore, there may be discrepancies in the findings across studies that administer multiple screening measures because each measure is based on different normative data. The key corollary here is that screening instruments may not capture the breadth and depth of emotional concerns as well as their associations with somatic and cognitive concerns in individuals with Long COVID. Using screening measures may therefore limit the ability to determine diagnosis, prognosis, and treatment recommendations in Long COVID.

Clinicians and researchers may benefit from using psychological measures that assess various subjective emotional, cognitive, and somatic concerns in individuals with Long COVID. The Personality Assessment Inventory (PAI; Morey, 1991) is among the most well-validated measures of psychological functioning that can be used for these purposes. The PAI not only captures a wide range of emotional, cognitive, and somatic concerns that are often not assessed with screening measures (e.g., delineating between physiological vs. affective vs. emotional aspects of depression and anxiety), but also offers normative data to understand whether such concerns are elevated in comparison to healthy or clinical samples. By reporting a broader range of psychological characteristics and including the severity of these characteristics compared to normative data, the PAI enables better analysis of the patient's presenting concerns and may lead to more targeted and effective treatments. Using the PAI vs. multiple screening measures can also promote more consistency and uniformity in the interpretation of patients' ratings. Unlike most screening instruments, this measure also provides empirically established indicators to flag whether someone is overreporting or underreporting symptoms in such a manner that would render their ratings non-credible and uninterpretable (Morey, 1991). Inclusion of these established indicators enhances reliability of the assessment, thus decreasing measurement bias.

To our knowledge, only two studies (Fry et al., 2023; Whiteside et al., 2022) have reported various emotional, cognitive, and somatic concerns in individuals with Long COVID using the PAI. Both studies included samples with predominately young adult patients who were referred to a post-COVID-19 clinic for clinical neuropsychological evaluation. Whiteside et al. (2022) found that most patients in their sample had clinically elevated depression and anxiety symptoms as well as somatic and cognitive concerns. They speculated that such concerns may be influenced by a vulnerability to psychological distress, premorbid somatic preoccupation influencing patients to seek medical attention, or socioenvironmental factors leading to somatic preoccupation. Consistent with other studies (Lemogne et al., 2023; Wang et al., 2022), these authors suggested that historical psychiatric symptoms might also be contributory. However, these studies did not extensively characterize the patients' historical mental health factors beyond diagnosis, omitting information related to past psychotherapy, pharmacotherapy, psychiatric hospitalization, suicide attempts, and trauma (Alkodaymi et al., 2022; Montani et al., 2022; Nalbandian et al., 2021; Oronsky et al., 2023; Marchi et al., 2023). Consideration of these historical mental health factors may further inform our understanding and treatment of psychological symptoms associated with Long COVID (Mikolić et al., 2019).

In summary, prior research has identified several emotional concerns in patients with Long COVID and noted that these concerns may influence the subjective experience of persistent somatic and cognitive symptoms following COVID-19. However, our understanding of these relationships is complicated by the number of studies relying on screening measures to assess emotional, cognitive, and somatic concerns in patients with Long COVID. To better understand these emotional, cognitive, and somatic concerns, researchers should use more comprehensive assessment measures and also consider the influence of predisposing mental health factors. Fry et al. (2023) and Whiteside et al. (2022) are the only studies that have addressed limitations and investigated the various subjective concerns in patients with Long COVID using comprehensive measures. However, they did not examine any predisposing mental health factors. As such, additional research is needed to expand upon their findings and consider the role of prior mental health factors.

### Current study

We sought to expand upon these studies by reporting various psychological characteristics and emotional, cognitive, and somatic concerns in a Long COVID sample. We also characterized these aspects of psychological functioning in the context of various historical mental health factors. Specifically, our aims were to (1) identify patients' emotional, somatic, and cognitive concerns using the PAI, and (2) examine such concerns in the context of the patients' mental health histories. Based on prior research, we hypothesized that (1) PAI scales related to depression, anxiety, somatic, and cognitive concerns would be most frequently elevated, and (2) the majority of patients would have a history of mental health diagnosis and treatment. Based on these initial descriptive findings, we offer some potential explanations regarding the role of preexisting mental health factors and implications for Long COVID mental health evaluation and treatment.

## Methods

### Participants and procedures

This cross-sectional descriptive pilot study is based on a sample of patients referred for additional neuropsychological screening following evaluation in a neurology COVID-19 clinic. The neurology COVID-19 clinic, from which the patients were referred, evaluates both hospitalized and non-hospitalized patients (the latter being the majority) who present with neurological symptoms following COVID-19, primarily those involving cognitive concerns (for details, see 3). All these patients met criteria for Long-COVID, presenting with ongoing symptoms ≥12 weeks post-COVID-19 symptom onset, with symptoms persisting for a minimum of 2 months (Soriano et al., 2022). Initial COVID-19 diagnosis was confirmed via polymerase chain reaction, serum testing, hospitalization report, or clinically via self-reported symptoms if diagnostic testing was not available at the time of their infection. The neurology COVID-19 clinic screened these patients with select neuropsychological measures from the electronic National Institute of Health Cognitive Toolbox (for details, see Perez Giraldo et al., 2023). Patients who scored one standard deviation below the mean of the normative population were referred for additional neuropsychological screening. This neuropsychological screening included a cognitive screener, neuropsychological clinical interview, and medical records review. The purpose of this neuropsychological screening was to determine if detailed and comprehensive neuropsychological assessment was indicated, and to identify any factors that may be contributing to the patients' cognitive concerns. These neuropsychological screenings were conducted by a board-certified clinical neuropsychologist. All patients consented for their clinical data to be used for research as part of the larger, IRB-approved study investigating the neurological correlates of COVID-19.

Twenty-eight patients underwent additional neuropsychological screening between 6/29/2021 and 12/21/2022, which included the administration of the PAI. We attempted to control for selection bias by including consecutive referrals between this timeframe. Of the 28 patients, two were excluded from the current study analyses because they demonstrated invalid symptom reporting as defined by empirical symptom validity indicators embedded within the PAI. The exact cutoffs used for these validity indicators have been validated in mixed neuropsychiatric samples (Hawes and Boccaccini, 2009; Morey, 2007). Scoring above these cutoffs suggests that an individual is likely responding in an inconsistent manner or in an overly negative manner, above and beyond what is expected for patients with genuine psychopathology. Elevated scores on any of these overreporting scales may render their symptom reporting invalid and uninterpretable. It is important to recognize that although these elevated scores indicate an unusually negative response style, they do not infer the reason or motivation for responding in such manner.

The final sample comprised 26 patients after removing those with invalid symptom reporting. These patients underwent neuropsychological screening evaluation on average 15 months after their initial COVID-19 symptom onset (*SD* = 7.48; range: 3–30 months). No participants had a history of persistent post-concussive symptoms or a serious neurologic disorder known to significantly impact their psychological functioning (stroke, epilepsy, or dementia); see below for details regarding data collection of past medical history. This study was not preregistered, and data are available upon request.

### Measures

The PAI, a well-validated psychological test for clinical evaluations, was used to assess psychological functioning (Martin et al., 2015; Kotov et al., 2017; Morey, 2007). This test comprises 344 items assessing self-reported emotional, somatic, and cognitive concerns as well as characterological traits known to impact treatment response and social support. It includes 22 non-overlapping scales categorized by four validity scales (Inconsistency, Infrequency, Negative Impression, and Positive Impression); 11 clinical scales (Somatic Complaints, Anxiety, Anxiety-Related Disorders, Depression, Mania, Paranoia, Schizophrenia, and Borderline Features, Antisocial Features, Alcohol Problems, and Drug Problems); five treatment scales (Aggression, Suicidal Ideation, Stress, Non-support, and Treatment Rejection); and two interpersonal scales (Dominance and Warmth).

The clinical scales can be broken into subscales that represent more specific descriptions of each construct. For instance, the Anxiety full scale is stratified by cognitive, affective, and physiological subscales, representing different dimensions of anxiety. Clinical scale elevations are determined by an average of the corresponding subscale elevations. Lastly, the PAI includes scales for an Alternative Model of Personality Disorders (e.g., Negative Affectivity, Detachment, Antagonism, Perseveration). Alternative scales were introduced to the PAI to capture a broader range of traits. These scales are designed to offer a more superordinate interpretation of psychopathology as compared to the use of individual scales in isolation (Hopwood et al., 2013). These alternative scales, while presented in the Results section, are still considered experimental and not a primary focus in our study. We presented them to support the development of the PAI. We provided a brief description of each elevated scale in the Results section, but for a full description of the PAI please see Morey (1991).

To draw comparisons to the known literature on Long COVID, we reported these scales based on their assessment of emotional, somatic, or cognitive concerns. We only reported scales that were clinically elevated, which is defined as full scale T-scores ≥70 as compared to a large healthy standardization sample (*N* > 3,000; Morey, 2007). We reported subscale and alternative scales that were deemed clinically relevant based on T-scores ≥65. Although these cutoffs are empirically established, we also conducted a frequency analysis of elevations based on more stringent cutoffs that are sometimes used in research to improve issues regarding reliability. For this supplemental frequency analysis, clinical elevations were defined by full scale T-scores ≥65 and subscale T-scores ≥70 (see Supplementary Table S1).

Preexisting mental health data were obtained via medical records review and a semi-structured, 1-h clinical interview. The semi-structured clinical interview asked about patients' presenting concerns (cognitive, emotional, somatic), prior neurologic work-up (neuroimaging results, neurocognitive testing, neurologic screenings), self-reported medical history, current medications, sleep, appetite, psychiatric history, substance abuse history, developmental and educational history, occupational history, and family history. To the extent possible, the information elicited during the clinical interview was referenced against the patients' medical records. A research assistant extracted these data that were documented in the clinical interview and the patients' medical records. Preexisting mental health data were defined as occurring before the onset of COVID-19. These data included patients' history of mental health disorders (presence/absence, and type), psychopharmacotherapy or psychotherapy (presence/absence), psychiatric hospitalization (presence/absence, and frequency), suicide attempts (presence/absence, frequency), and abuse (presence/absence, frequency, and type). Criteria for mental health disorder diagnosis was explicitly discussed with patients during the clinical interview and based on the *Diagnostic and Statistical Manual of Mental Disorders-Fifth Edition* (American Psychiatric Association, 2013). To better contextualize participants' data, we also reported their demographics (age, sex, race, and education) and performance on a brief cognitive screener (Montreal Cognitive Assessment; MoCA), which were collected during the neuropsychological screening.

## Results

### Demographics

As seen in Table 1, most patients were females (85%) in their mid-40s who identified as White (81%) and had an average of 16 years of educational attainment. Most patients (85%) were not hospitalized for COVID-19 and had an average MoCA score of 26/30 at the time of their evaluation.

**Table 1:** Demographics and sample characteristics (*N* = 26).

**Characteristic**	**Mean (standard deviation; range) Frequency (proportion)**
Age	*M* = 46.19 (11.68; 29–73)
Education	*M* = 16.31 (1.72; 12–19)
Female sex	22 (84.6%)
**Racial identity**	
Non-Hispanic white	21 (80.8%)
Non-hispanic black	3 (11.5%)
Asian	1 (3.8%)
Hispanic	2 (7.7%)
MoCA score	*M =* 26.41 (2.46; 20–30)
History of COVID-19 hospitalization	4 (15.4%)
History of psychotherapy	22 (84.6%)
History of psychotropic medications	13 (50.0%)
History of psychiatric hospitalization	3 (11.5%)
History of suicide attempt	4 (15.4%)
History of mental health disorder diagnosis Any mental health disorder	21 (80.8%)
Anxiety disorder	15 (57.7%)
Depression disorder	14 (53.8%)
PTSD or acute stress-related disorder	8 (30.8%)
Attention-deficit/hyperactivity disorder	4 (15.4%)
Adjustment disorder	1 (3.8%)
Personality disorder	1 (3.8%)
Bipolar 2 disorder	1 (3.8%)
**History of abuse**	
Any form of abuse	15 (57.7%)
Physical abuse	5 (19.2%)
Emotional abuse	9 (34.6%)
Sexual abuse	9 (34.6%)

COVID-19, Coronavirus Disease; MoCA, Montreal Cognitive Assessment; PTSD, Post-Traumatic Stress Disorder.

### Overview of emotional, cognitive, and somatic concerns

The frequency and description of each PAI elevations are described below. Specifically, we described the full scales, subscales, and alternative scales according to whether they assess somatic, cognitive, or emotional concerns. Overall, most patients exhibited multiple elevations across the full scales, subscales, and alternative scales, as defined by full scale T-scores ≥70 and subscale and alternative scale T-scores ≥65. Only three patients did not exhibit any elevated scales. On average, patients exhibited 1.77 full scale elevations (excluding validity scales; *SD* = 1.92, range: 0–8), 5.92 subscale elevations (*SD* = 4.62, range: 0–19), and 1.0 alternative scale elevations (*SD* = 1.73, range: 0–6). Minimal differences were observed in the rate of elevations when full scale T-scores ≥65 and subscale T-scores ≥70 were used as the elevation cutoffs (see Supplementary Table S1).

### Somatic concerns

The Somatic Complaints (full) scale evaluates concerns about physical health symptoms. As seen in Table 2, this was the most frequently elevated scale (62%), and all the Somatic Complaints subscales were elevated. Approximately 62 and 65% of patients had elevated scores on the Somatization and Health Concerns subscales, respectively, while 54% had elevations on the Conversion subscale. As with the other subscales, these subscales assess for distinct, non-overlapping concerns. The Health Concerns subscale assesses general worries about health, Somatization assesses preoccupation with physical ailments, and Conversion assesses functional difficulties due to symptoms associated with functional neurological disorder.

**Table 2:** Full scale and subscale personality assessment inventory elevations (*N* = 26).

**PAI full scales and subscales**	**Scale elevation frequency (proportion)**
**Scales assessing somatic concerns**	
Somatic concerns	61.5% (16)
Conversion	53.8% (14)
Somatization	61.5% (16)
Health concerns	65.4% (17)
**Scales assessing cognitive concerns**	
Schizophrenia	3.8% (1)
Psychotic experiences	3.8% (1)
Social detachment	11.5% (3)
Thought disorder	50.0% (13)
**Scales assessing emotional concerns**	
Anxiety	26.9% (7)
Cognitive	23.1% (6)
Affective	34.6% (9)
Physiological	27.0% (7)
Anxiety-related disorders	15.4% (4)
Obsessive-compulsive	15.4% (4)
Phobias	15.4% (4)
Traumatic stress	19.2% (5)
Depression	42.3% (11)
Cognitive	27.0% (7)
Affective	30.8% (8)
Physiological	57.7% (15)
Mania	0.0% (0)
Activity level	0.0% (0)
Grandiosity	11.5% (3)
Irritability	0.0% (0)
Paranoia	0.0% (0)
Hypervigilance	3.8% (1)
Persecution	3.8% (1)
Resentment	0.0% (0)
Borderline	3.8% (1)
Affective instability	19.2% (5)
Identity problems	11.5% (3)
Negative relationships	11.5% (3)
**Scales assessing treatment-related behaviors**	
Self-harm	11.5% (3)
Antisocial features	0.0% (0)
Antisocial behaviors	7.7% (2)
Egocentricity	0.0% (0)
Stimulus-seeking	0.0% (0)
Alcohol problems	0.0% (0)
Drug problems	0.0% (0)
Aggression	0.0% (0)
Aggressive attitude	7.7% (2)
Verbal aggression	3.8% (1)
Physical aggression	3.8% (1)
Suicidal ideation	7.7% (2)
Stress	15.4% (4)
Non-support	3.8% (1)
Treatment rejection	0.0% (0)
**Scales assessing interpersonal features**	
Dominance	3.8% (1)
Warmth	0.0% (0)

PAI, Personality Assessment Inventory. Full scales were considered elevated is they had T-scores ≥70, whereas subscales were considered elevated if they had T-scores ≥65.

### Cognitive concerns

Cognitive concerns regarding attention lapses and concentration difficulties were measured primarily using the Thought Disorders subscale of the Schizophrenia full scale (Morey, 1991). The Thought Disorders subscale was elevated in 50% of the sample, while the Schizophrenia (full) scale was only elevated in <4% of the sample. However, this discrepancy is not unexpected since the Schizophrenia scale should be interpreted by its subscales rather than as a unitary scale (Morey, 1991). No patients had elevations on the other Schizophrenia subscales. Similar to the Thought Disorder findings, the most commonly elevated alternative scale was Distractibility, which assesses one's perceived concentration difficulties.

### Emotional concerns

Depression (42%) and Anxiety (27%) were the most commonly elevated full scales pertaining to emotional concerns. All the Depression subscales were elevated, with the Physiological subscale showing the highest elevations compared to the Cognitive and Affective subscales. The Cognitive subscale assesses depressive thoughts (e.g., “I am worthless”), Affective assesses depressive feelings (e.g., sadness), and Physiological assesses depressive symptoms like fatigue or loss of appetite. All the Anxiety subscales were similarly elevated. The Anxiety scale assesses generalized anxiety symptoms, whereas the Anxiety-Related Disorders scale assesses symptoms specific to certain disorders (e.g., presentation of anxiety in obsessive-compulsive disorder) and is best interpreted by its subscales rather than as a unitary scale (Morey, 1991). In comparison to the Anxiety subscale, there were fewer elevations on the Anxiety-Related Disorder subscales relating to trauma, obsessive-compulsive, and phobia disorders.

Approximately 19% of the sample had elevated scores on the Affective Instability subscale, which is within the Borderline full scale, suggesting difficulty with emotion regulation. The Emotional Lability subscale, which also assesses aspects of emotion dysregulation, was elevated in 12% of the sample. In addition to the clinical scales, 12% of the sample had an elevated Stress scale, indicating that these individuals were experiencing significant psychosocial stressors. Furthermore, 12% of the sample produced elevated Perseveration, Anhedonia, and Depressivity alternative scales. The Perseveration subscale assesses the tendency to repeat or “get stuck” on a thought that is typically distressing. The Anhedonia subscale assesses difficulty experiencing pleasure and joy and the Depressivity subscale assesses for feelings of sadness.

### Past psychiatric history

Prior to their COVID-19 infection, 85% of the patients received psychotherapy, 42% received psychotropic medications, 12% were hospitalized for psychiatric reasons, and 15% attempted suicide at least once in their lifetime. Approximately 81% of the sample had a psychiatric diagnosis prior to COVID-19, with anxiety, depression, post-traumatic stress disorder (PTSD), and attention-deficit/hyperactivity disorder being the most common. Additionally, over half of the sample disclosed some form of psychological trauma, mostly involving sexual abuse.

## Discussion

This study is among the first to provide a detailed report of the emotional, cognitive, and somatic concerns, along with relevant historical mental health factors, in a clinically referred sample of patients with neurologic symptoms following Long COVID. Findings replicate prior studies demonstrating that patients with Long COVID have high rates of somatic and cognitive concerns as well as depression and anxiety, as compared to normative data based on neurotypical samples. However, it is important to note that the current findings should be interpreted with caution given the exploratory and pilot nature of the study as well as the absence of a control group.

### Somatic concerns

All somatic concern subscales (conversion, somatization, health concerns) were elevated in our sample, which is unsurprising given that patients were referred from a neurology COVID-19 clinic. These findings are also consistent with previous Long COVID literature (Horn et al., 2021; Willis and Chalder, 2021). Whiteside et al. (2022) and Fry et al. (2023) found that nearly half of their sample had elevated somatic concerns on the PAI, which were primarily driven by health-related concerns. They hypothesized that health-related concerns might prompt individuals to seek medical care; however, this may be biased considering that their studies (like ours) were investigating clinical as opposed to community samples. Compared to community samples, clinically referred samples may include individuals with potentially worse symptoms, those who are privier to changes in their health, as well as those who have better access to healthcare resources. Whiteside et al. (2022) and Fry et al. (2023) also proposed that individuals who are more vigilant of health-related changes might find persistent symptoms more bothersome than others. Somatic concerns are ubiquitous in medical populations (Goldberg and McGee, 2011), but elevated levels of conversion in addition to somatization and health-related anxiety may suggest an overlay with functional neurological disorders (Mavroudis et al., 2023; Picon et al., 2021). The potential transdiagnostic features in functional neurological disorders and Long COVID may be relevant for future research investigation but are beyond the scope of this paper.

### Cognitive concerns

The Thought Disorder subscale was the only elevated Schizophrenia subscale in our sample. This scale should be interpreted in isolation, as it is indicative of cognitive concerns rather than psychotic symptoms, which is further supported by the elevated Distractibility scale (Morey, 1991). These elevations were also expected given that patients were referred for further screening due to neurologic symptoms often involving cognitive concerns. Both Whiteside et al. (2022) and Fry et al. (2023) observed a similar elevation and also attributed it to cognitive concerns. It has been well described in the literature that patients with Long COVID report “brain fog” and related cognitive concerns (Graham et al., 2021; Ceban et al., 2022). It has been demonstrated in other neuropsychiatric samples that reported cognitive concerns are typically more severe than compared to impairments on objective cognitive testing (Stillman et al., 2020). Most research on this discrepancy suggests that perceived cognitive impairment is more indicative of depression, anxiety, and somatic concerns rather than actual cognitive ability (Finley et al., 2023; Oyesanya et al., 2020). However, it is possible that standardized testing may not capture subtle cognitive changes. Patients in our sample may have been experiencing mild cognitive sequalae from COVID-19. It is also possible that depression, anxiety, and somatic concerns were driving some of these cognitive concerns, as all patients with cognitive concerns had elevated emotional and somatic concerns. Alternatively, cognitive concerns may increase the propensity for emotional concerns (Oyesanya et al., 2020). Nonetheless, we cannot fully discern the underlying reasons for cognitive concerns, nor the relationship to emotional concerns, in this sample without further investigation. Although every patient within our sample performed one standard deviation below the mean on one subtest of a digitized cognitive screening measure (i.e., National Institute of Health Toolbox), this was documented several months prior to our study evaluation, and they performed within expectation on the MoCA (average MoCA score was 26/30) during the current study evaluation (Islam et al., 2023). Finally, neither the MoCA nor the initial screening measure include well-validated indicators of performance validity to establish the veracity of their cognitive performance. Although the PAI includes validity indicators, recent research suggests that validity indicators embedded within self-report measures should not be used to determine performance validity in neurocognitive testing (Finley, 2024; Finley et al., 2023).

### Current emotional concerns

Consistent with previous literature (Bourmistrova et al., 2022; Méndez et al., 2021), our sample reported significant current emotional concerns related to depression, anxiety, and stress. Symptoms associated with these concerns, however, varied widely. Patients generally expressed the greatest concern for physiological symptoms of depression, with less variation observed in anxiety symptoms. Elevations in physiological depression pose the question of whether such symptoms, like fatigue, stem from depression, systemic effects of COVID-19, or both. The elevations in physiological depression may also be due to comorbid medical or psychiatric conditions. Based on the high rates of elevated somatic concerns, it is not surprising that the physiological aspects of depression were also elevated. However, it was unexpected to find that patients did not report similar rates of concerns regarding the physiological aspects of anxiety. Whiteside et al. (2022) and Fry et al. (2023) also noted elevated depression and anxiety concerns in their Long COVID samples, and similarly found that physiological depression symptoms were commonly elevated. Another trend observed in their studies and ours was that about a fifth of patients with Long COVID had elevated concerns regarding traumatic stress. This finding aligns with the literature demonstrating high rates of PTSD and acute traumatic stress in patients with Long COVID (Nalbandian et al., 2021). These distinctions in emotion-related concerns may have different effects on treatment outcomes and adherence. For example, it has been shown that hopelessness within depression is associated with poorer response to antidepressant medication (Papakostas et al., 2007). Further, difficult-to-treat depression is associated with features such as hopelessness, anhedonia and low self-esteem and may require more nuanced treatment approaches (Casey et al., 2013). Lastly, patients with physiological symptoms (e.g., fatigue) may feel physically incapable of attending treatment, thus creating a barrier to care (Trivedi, 2004).

### Psychiatric history

In regard to psychiatric history, most patients in our sample had received a mental health disorder diagnosis and psychiatric treatment prior to COVID-19. Several patients reported a history of suicide attempts, psychiatric hospitalizations, and abuse. Whiteside et al. (2022) found that 63% of their sample had at least one historical psychiatric diagnosis, primarily depression, followed by anxiety and PTSD. They found that patients with premorbid psychiatric conditions exhibited more emotional distress on the PAI, suggesting that prior psychiatric history may have partially influenced the high rates of elevated concerns in patients with Long COVID.

Our findings are consistent with the existing literature regarding the high prevalence of prior psychiatric history in those with Long COVID. Further, there is a growing body of evidence that preexisting psychiatric history is a risk factor for Long COVID, yet the nature of this relationship remains uncertain (Lemogne et al., 2023; Wang et al., 2022). As hypothesized in other populations with persistent syndromes (Silverberg and Mikolić, 2023), psychiatric symptoms could be a reaction to Long COVID or a precursor that increases the risk of reporting such symptoms. That said, it must be noted that the psychological symptoms gleaned from the PAI may reflect symptomology from comorbid conditions, as opposed to Long COVID. Further research is required to determine this relationship.

### Treatment implications

Elucidating the potential temporally reciprocal association between psychiatric history and psychological characteristics in Long COVID remains an ongoing challenge (Cheng et al., 2023). Discerning these factors, however, does not seem possible without utilizing a comprehensive psychological test (such as the PAI) and conducting a thorough record review (including medical, academic, and prior [neuro]psychological evaluation as needed) and clinical diagnostic interview. These assessment methods allowed us to identify nuances and heterogeneity in psychological concerns among patients referred for the same health condition.

There are well-proposed, psychological interventions used to address emotional, cognitive, and somatic symptoms and concerns in patients with other persistent syndromes, such as persistent post-concussive syndrome, chronic fatigue syndrome, and fibromyalgia (Afari and Buchwald, 2003; Kennedy and Felson, 1996); these interventions may also apply to Long COVID. However, discussion of these approaches is beyond the scope of this paper.

Of note, it may be important to provide education on expected symptom recovery as a proactive (as opposed to reactive) to preventing Long COVID symptoms. Our study, along with existing literature suggests that individuals with preexisting mental health symptoms are at risk for persistent psychological symptoms following SARS-CoV-2 infection. SARS-CoV-2 has not been eradicated and is likely to persist, suggesting potential for a proactive public health intervention. Further investigation is required, but we suggest movement toward early education for patients presenting with COVID-19 and premorbid psychiatric risk factors regarding potential sequelae.

### Limitations

The primary limitation is that our study is descriptive in nature and lacks comparison to a control group. Without formal statistical analysis using a comparative group, we cannot confirm the extent to which the severity and frequencies of symptoms reported in our sample significantly differ from the normative population or other clinical populations. However, we did use normative scores gleaned from the PAI, which allows for comparison to other psychiatric conditions. A similar limitation is that we only included reports from a small sample of patients who were referred for neuropsychological screening based on neurologic symptoms involving cognitive concerns, which limits the generalizability of the findings. Our sample also lacked ethno-racial diversity. Ethno-racially minoritized individuals with Long COVID may not seek clinical treatment due to systemic inequities impacting financial and health-related disparities. Indeed, this may explain why our sample was lacking ethno-racial diversity. However, the neurology COVID-19 clinic, where patients were referred from, attempted to mitigate these barriers by not requiring physician referrals for evaluation and by accommodating patients either in-person or in telehealth visits. Nevertheless, our study findings may not be applicable to those who do not fit the ethno-racial makeup of our sample. Additionally, socioeconomic status was not included in the demographic data reported for our patient sample, thus limiting our ability to determine the applicability of study findings to populations of lower socioeconomic status. Future research should use statistical analysis to compare a larger, more diverse sample of patients with Long COVID to a control group. Addressing these potential biases will be necessary to improve the generalizability of the current study findings.

Another limitation is that we included patients who were evaluated at various time points, ranging from 3 to 30 months post-COVID-19 symptom onset, but were unable to control for the potentially confounding effect of symptom duration on the severity of psychological concerns. Future research should control for potential confounding effects such as duration of symptoms. Further, although we provided mental health history preceding participants' COVID-19, we are still unable to fully elucidate the temporally reciprocal relationship between psychological concerns gleaned from the PAI and COVID-19. Thus, it is possible that prior psychiatric conditions independently led to elevations in the PAI. A prospective design that incorporates pre- and post-COVID-19 PAI assessment is needed to examine the extent to which mental health history increases the risk of persistent psychological concerns in patients with Long COVID. If future studies wish to address the aforementioned limitations, statistical modeling will be necessary. It would also be helpful to consider the actual scores from each of the PAI full scales and subscales, rather than a binary elevated score, in future statistical analyses.

## Conclusion

These findings suggest that in addition to somatic and cognitive concerns, patients with Long COVID may exhibit extensive psychiatric history and emotional concerns that are unlikely to be detected with mood screeners. Consideration of prior psychiatric history and utilization of comprehensive assessments like the PAI allows for a comprehensive, and potentially patient-centered approach that may inform care for patients with Long COVID.

## Data Availability

The raw data supporting the conclusions of this article will be made available by the authors, without undue reservation.

## References

[B1] Afari N. Buchwald D. (2003). Chronic fatigue syndrome: a review. Am. J. Psychiatry 160, 221–236. 10.1176/appi.ajp.160.2.221 12562565

[B2] Alkodaymi M. S. Omrani O. A. Ashraf N. Shaar B. A. Almamlouk R. Riaz M.. (2022). Prevalence of post-acute COVID-19 syndrome symptoms at different follow-up periods: a systematic review and meta-analysis. Clin. Microbiol. Infect. 28, 657–666 10.1016/j.cmi.2022.01.014 35124265 PMC8812092

[B3] American Psychiatric Association. (2013). Diagnostic and Statistical Manual of Mental Disorders (5th edn.). Washington, DC. 10.1176/appi.books.9780890425596

[B4] Bourmistrova N. W. Solomon T. Braude P. Strawbridge R. Carter B. (2022). Long-term effects of COVID-19 on mental health: a systematic review. J. Affect. Disord. 299, 118–125. 10.1016/j.jad.2021.11.031 34798148 PMC8758130

[B5] Cabrera Martimbianco A. L. Pacheco R. L. Bagattini Â. M. Riera R. (2021). Frequency, signs and symptoms, and criteria adopted for long COVID-19: a systematic review. Int. J. Clin. Pract. 75: e14357. 10.1111/ijcp.14357 33977626 PMC8236920

[B6] Cahan J. Finley J. A. Cotton E. Orban Z. S. Jimenez M. Weintraub S.. (2024). Cognitive functioning in patients with neuro-PASC: the role of fatigue, mood, and hospitalization status. Front. Neurol. 15:1401796. 10.3389/fneur.2024.1401796 38994492 PMC11236596

[B7] Carfì A. Bernabei R. Landi F. Gemelli against COVID-19 Post-Acute Care Study Group. (2020). Persistent symptoms in patients after acute COVID-19. JAMA 324, 603–605. 10.1001/jama.2020.12603 32644129 PMC7349096

[B8] Casey M. F. Perera D. N. Clarke D. M. (2013). Psychosocial treatment approaches to difficult-to-treat depression. Med. J. Aust. 199, S52–S55. 10.5694/mja12.10629 25370289

[B9] CDC (2023). Long COVID—household pulse survey—COVID-19. CDC. Available online at: https://www.cdc.gov/nchs/covid19/pulse/long-covid.htm

[B10] Ceban F. Ling S. Lui L. M. W. Lee Y. Gill H. Teopiz K. M.. (2022). Fatigue and cognitive impairment in Post-COVID-19 syndrome: a systematic review and meta-analysis. Brain Behav. Immun. 101, 93–135. 10.1016/j.bbi.2021.12.020 34973396 PMC8715665

[B11] Cheng A. L. Anderson J. Didehbani N. Fine J. S. Fleming T. K. Karnik R.. (2023). Multi-disciplinary collaborative consensus guidance statement on the assessment and treatment of mental health symptoms in patients with post-acute sequelae of SARS-CoV-2 infection (PASC). PM R 15, 1588–1604. 10.1002/pmrj.13085 37937672

[B12] Delgado-Alonso C. Valles-Salgado M. Delgado-Álvarez A. Gómez-Ruiz N. Yus M. Polidura C.. (2022). Examining association of personality characteristics and neuropsychiatric symptoms in post-COVID syndrome. Brain Sci. 12:265. 10.3390/brainsci12020265 35204028 PMC8870488

[B13] Engelmann P. Reinke M. Stein C. Salzmann S. Löwe B. Toussaint A.. (2024). Psychological factors associated with Long COVID: a systematic review and meta-analysis. EClinicalMedicine 74:102756. 10.1016/j.eclinm.2024.102756 39764180 PMC11701445

[B14] Finley J. A. Cerny B. M. Brooks J. M. Obolsky M. A. Haneda A. Ovsiew G. P.. (2023). Cross-validating the clinical assessment of attention deficit-adult symptom validity scales in adults with attention deficit/hyperactivity disorder. J. Clin. Exp. Neuropsychol. 46, 111–123. 10.1080/13803395.2023.2283940 37994688

[B15] Finley J. C. A. (2024). Performance validity testing: the need for digital technology and where to go from here. Front. Psychol. 15:1452462. 10.3389/fpsyg.2024.1452462 39193033 PMC11347285

[B16] Fry L. Logemann A. Waldron E. Holker E. Porter J. Eskridge C.. (2023). Emotional functioning in long COVID: comparison to post-concussion syndrome using the personality assessment inventory. Clin. Neuropsychol. 38, 963–983. 10.1080/13854046.2023.2264546 37838973

[B17] Galvez-Sánchez C. M. Reyes Del Paso G. A. Duschek S. (2018). Cognitive impairments in fibromyalgia syndrome: associations with positive and negative affect, alexithymia, pain catastrophizing and self-esteem. Front. Psychol. 9:377. 10.3389/fpsyg.2018.00377 29623059 PMC5874325

[B18] Goldberg D. S. McGee S. J. (2011). Pain as a global public health priority. BMC Public Health 11:770. 10.1186/1471-2458-11-770 21978149 PMC3201926

[B19] Graham E. L. Clark J. R. Orban Z. S. Lim P. H. Szymanski A. L. Taylor C.. (2021). Persistent neurologic symptoms and cognitive dysfunction in non-hospitalized COVID-19 “long haulers”. Ann. Clin. Transl. Neurol. 8, 1073–1085. 10.1002/acn3.51350 33755344 PMC8108421

[B20] Hawes S. W. Boccaccini M. T. (2009). Detection of overreporting of psychopathology on the personality assessment inventory: a meta-analytic review. Psychol. Assess. 21, 112–124. 10.1037/a0015036 19290771

[B21] Hopwood C. J. Wright A. G. Krueger R. F. Schade N. Markon K. E. Morey L. C. (2013). DSM−5 pathological personality traits and the personality assessment inventory. Assessment 20, 269–285. 10.1177/1073191113486286 23610235

[B22] Horn M. Fovet T. Vaiva G. D'Hondt F. Amad A. (2021). Somatic symptom disorders and long COVID: a critical but overlooked topic. Gen. Hosp. Psychiatry 72, 149–150. 10.1016/j.genhosppsych.2021.06.007 34215435 PMC8233051

[B23] Islam N. Hashem R. Gad M. Brown A. Levis B. Renoux C.. (2023). Accuracy of the montreal cognitive assessment tool for detecting mild cognitive impairment: a systematic review and meta-analysis. Alzheimers Dement. 19, 3235–3243. 10.1002/alz.13040 36934438

[B24] Kennedy M. Felson D. T. (1996). A prospective long-term study of fibromyalgia syndrome. Arthritis Rheum. 39, 682–685. 10.1002/art.1780390422 8630121

[B25] Kotov R. Krueger R. F. Watson D. Achenbach T. M. Althoff R. R. Bagby R. M.. (2017). The hierarchical taxonomy of psychopathology (HiTOP): a dimensional alternative to traditional nosologies. J. Abnorm. Psychol. 126, 454–477. 10.1037/abn0000258 28333488

[B26] Kung B. Chiang M. Perera G. Pritchard M. Stewart R. (2021). Identifying subtypes of depression in clinician-annotated text: a retrospective cohort study. Sci. Rep. 11:22426. 10.1038/s41598-021-01954-4 34789827 PMC8599474

[B27] Lemogne C. Gouraud C. Pitron V. Ranque B. (2023). Why the hypothesis of psychological mechanisms in long COVID is worth considering. J. Psychosom. Researc. 165: 2111135. 10.1016/j.jpsychores.2022.111135 36623391 PMC9825049

[B28] Marchi M. Grenzi P. Serafini V. Capoccia F. Rossi F. Marrino P.. (2023). Psychiatric symptoms in long-COVID patients: a systematic review. Front. Psychiatry 14:1138389. 10.3389/fpsyt.2023.1138389 37415689 PMC10320160

[B29] Martin P. K. Schroeder R. W. Odland A. P. (2015). Neuropsychologists validity testing beliefs and practices: a survey of north American professionals. Clin. Neuropsychol. 29, 741–776. 10.1080/13854046.2015.1087597 26390099

[B30] Mavroudis I. Chatzikonstantinou S. Petridis F. Palade O. D. Ciobica A. Balmus I. M. (2023). Functional overlay model of persistent post-concussion syndrome. Brain Sci. 13:1028. 10.3390/brainsci13071028 37508960 PMC10377031

[B31] Méndez R. Balanzá-Martínez V. Luperdi S. C. Estrada I. Latorre A. González-Jiménez P.. (2021). Short-term neuropsychiatric outcomes and quality of life in COVID-19 survivors. J. Intern. Med. 290, 621–631. 10.1111/joim.13262 33533521 PMC8013333

[B32] Mikolić A. Polinder S. Retel Helmrich I. R. A. Haagsma J. A. Cnossen M. C. (2019). Treatment for posttraumatic stress disorder in patients with a history of traumatic brain injury: a systematic review. Clin. Psychol. Rev. 73:101776. 10.1016/j.cpr.2019.101776 31707182

[B33] Montani D. Savale L. Noel N. Meyrignac O. Colle R. Gasnier M.. (2022). Post-acute COVID-19 syndrome. Eur. Respir. Rev. 31:210185. 10.1183/16000617.0185-2021 35264409 PMC8924706

[B34] Morey L. C. (1991). Personality Assessment Inventory (PAI). Professional manual. Odessa, FL: Psychological Assessment Resources.

[B35] Morey L. C. (2007). Personality Assessment Inventory (PAI). Professional Manual (2nd ed.). Odessa, FL: Psychological Assessment Resources.

[B36] Nalbandian A. Sehgal K. Gupta A. Madhavan M. V. McGroder C. Stevens J. S.. (2021). Post-acute COVID-19 syndrome. Nat. Med. 27, 601–615. 10.1038/s41591-021-01283-z 33753937 PMC8893149

[B37] Newson J. J. Hunter D. Thiagarajan T. C. (2020). The heterogeneity of mental health assessment. Front. Psychiatry 11:76. 10.3389/fpsyt.2020.00076 32174852 PMC7057249

[B38] Oronsky B. Larson C. Hammond T. C. Oronsky A. Kesari S. Lybeck M.. (2023). A review of persistent post-COVID syndrome (PPCS). Clin. Rev. Allergy Immunol. 64, 66–74. 10.1007/s12016-021-08848-3 33609255 PMC7896544

[B39] Oyesanya M. Harmer C. J. Young A. H. (2020). Editorial: cognition in mood disorders. Front. Neurol. 10:1013. 10.3389/fpsyt.2019.01013 32082197 PMC7005635

[B40] Papakostas G. I. Petersen T. Homberger C. H. Green C. H. Smith J. Alpert J. E.. (2007). Hopelessness as a predictor of non-response to fluoxetine in major depressive disorder. Ann. Clin. Psychiatry. 19, 5–8. 10.1080/10401230601163451 17453655

[B41] Perez Giraldo G. S. Ali S. T. Kang A. K. Patel T. R. Budhiraja S. Gaelen J. I.. (2023). Neurologic manifestations of long COVID differ based on acute COVID-19 severity. Ann. Neurol. 94, 146–159. 10.1002/ana.26649 36966460 PMC10724021

[B42] Picon E. L. Perez D. L. Burke M. J. Debert C. T. Iverson G. L. Panenka W. J.. (2021). Unexpected symptoms after concussion: potential links to functional neurological and somatic symptom disorders. J. Psychosom. Res. 151:110661. 10.1016/j.jpsychores.2021.110661 34739941

[B43] Renna M. E. Quintero J. M. Fresco D. M. Mennin D. S. (2017). Emotion regulation therapy: a mechanism-targeted treatment for disorders of distress. Front. Psychol. 8:98. 10.3389/fpsyg.2017.00098 28220089 PMC5292405

[B44] Schretlen D. J. Finley J. C. A. Del Bene V. A. Varvaris M. (2024). The ubiquity of cognitive impairment in human illness: a systematic review of meta-analyses. Arch. Clin. Neuropsychol. 40:acae113. 10.1093/arclin/acae113 39667720

[B45] Serafini G. Costanza A. Aguglia A. Amerio A. Placenti V. Magnani L.. (2023). Overall goal of cognitive-behavioral therapy in major psychiatric disorders and suicidality: a narrative review. Med. Clin. North Am. 107, 143–167. 10.1016/j.mcna.2022.05.006 36402496

[B46] Silverberg N. D. Mikolić A. (2023). Management of psychological complications following mild traumatic brain injury. Curr. Neurol. Neurosci. Rep. 23, 49–58. 10.1007/s11910-023-01251-9 36763333

[B47] Soriano J. B. Murthy S. Marshall J. C. Relan P. Diaz J. V. WHO Clinical Case Definition Working Group on Post-COVID-19 Condition. (2022). A clinical case definition of post-COVID-19 condition by a Delphi consensus. Lancet Infect. Dis. 22, e102–e107. 10.1016/S1473-3099(21)00703-9 34951953 PMC8691845

[B48] Stillman A. M. Madigan N. Torres K. Swan N. Alexander M. P. (2020). Subjective cognitive complaints in concussion. J. Neurotrauma 37, 305–311. 10.1089/neu.2018.5925 31407632

[B49] Szuhany K. L. Simon N. M. (2022). Anxiety disorders: a review. JAMA 328, 2431–2445. 10.1001/jama.2022.22744 36573969

[B50] Taquet M. Geddes J. R. Husain M. Luciano S. Harrison P. J. (2021). 6-month neurological and psychiatric outcomes in 236 379 survivors of COVID-19: a retrospective cohort study using electronic health records. Lancet Psychiatry 8, 416–427. 10.1016/S2215-0366(21)00084-5 33836148 PMC8023694

[B51] Thase M. E. (2013). The multifactorial presentation of depression in acute care. J. Clin. Psychiatry 74, 3–8. 10.4088/JCP.12084su1c.01 24191971

[B52] Trivedi M. H. (2004). The link between depression and physical symptoms. Prim. Care Companion J. Clin. Psychiatry 6, 12–16. 16001092 PMC486942

[B53] van Gils A. Stone J. Welch K. Davidson L. R. Kerslake D. Caesar D.. (2020). Management of mild traumatic brain injury. Pract. Neurol. 20, 213–221. 10.1136/practneurol-2018-002087 32273394

[B54] Wang S. Quan L. Chavarro J. E. Slopen N. Kubzansky L. D. Koenen K. C.. (2022). Associations of depression, anxiety, worry, perceived stress, and loneliness prior to infection with risk of post-COVID-19 conditions. JAMA Psychiatry 79, 1081–1091. 10.1001/jamapsychiatry.2022.2640 36069885 PMC9453634

[B55] Whiteside D. M. Naini S. M. Basso M. R. Waldron E. J. Holker E. Porter J.. (2022). Outcomes in post-acute sequelae of COVID-19 (PASC) at 6 months post-infection part 2: psychological functioning. Clin. Neuropsychol. 36, 829–847. 10.1080/13854046.2022.2030411 35098861

[B56] Willis C. Chalder T. (2021). Concern for COVID-19 cough, fever and impact on mental health. What about risk of Somatic Symptom Disorder? J. Ment. Health 30, 551–555. 10.1080/09638237.2021.1875418 33522343

[B57] Zimmerman M. (2024). The value and limitations of self-administered questionnaires in clinical practice and epidemiological studies. World Psychiatry 23, 210–212. 10.1002/wps.21191 38727038 PMC11083863

