# Supplementary material for: Beyond mood screening: a pilot study of emotional, cognitive, and somatic concerns in patients with Long COVID
Source: Front Psychol. 2025 Jun 17;16:1517299. doi: 10.3389/fpsyg.2025.1517299 (PMC12209208; doi:10.3389/fpsyg.2025.1517299)
Supplement: Supplementary file 1 [file Table_1.docx]

| **Supplementary Table 1**  *Full scale and subscale Personality Assessment Inventory elevations (N = 26).* | |  |
| --- | --- | --- |
| PAI Full Scales (≥65) and Subscales (≥70) | Scale Elevation Frequency (Proportion) |  |
| **Scales Assessing Somatic Concerns** | | |
| Somatic Concerns | | 69.2% (18) |
| Conversion | | 42.3% (11) |
| Somatization | | 46.2% (12) |
| Health Concerns | | 53.8% (14) |
| **Scales Assessing Cognitive Concerns** | | |
| Schizophrenia | | 11.5% (3) |
| Psychotic Experiences | | 3.8% (1) |
| Social Detachment | | 7.7% (2) |
| Thought Disorder | | 50.0% (13) |
| **Scales Assessing Emotional Concerns** | |  |
| Anxiety | 38.5% (10) |  |
| Cognitive | 23.1% (6) |  |
| Affective | 30.8% (8) |  |
| Physiological | 11.5% (3) |  |
| Anxiety-Related Disorders | 23.1% (6) |  |
| Obsessive-Compulsive | 0.0% (0) |  |
| Phobias | 11.5% (3) |  |
| Traumatic Stress | 15.4% (4) |  |
| Depression | 46.2% (12) |  |
| Cognitive | 23.1% (6) |  |
| Affective | 23.1% (6) |  |
| Physiological | 30.8% (8) |  |
| Mania | 0.0% (0) |  |
| Activity Level | 0.0% (0) |  |
| Grandiosity | 11.5% (3) |  |
| Irritability | 0.0% (0) |  |
| Paranoia | 0.0% (0) |  |
| Hypervigilance | 3.8% (1) |  |
| Persecution | 0.0% (0) |  |
| Resentment | 0.0% (0) |  |
| Borderline | 7.7% (2) |  |
| Affective Instability | 11.5% (3) |  |
| Identity Problems | 7.7% (2) |  |
| Negative Relationships | 3.8% (1) |  |
| **Scales Assessing Treatment-Related Behaviors** | |  |
| Self-Harm | 11.5% (3) |  |
| Antisocial Features | 0.0% (0) |  |
| Antisocial Behaviors | 7.7% (2) |  |
| Egocentricity | 0.0% (0) |  |
| Stimulus-Seeking | 0.0% (0) |  |
| Alcohol Problems | 0.0% (0) |  |
| Drug Problems | 0.0% (0) |  |
| Aggression | 0.0% (0) |  |
| Aggressive Attitude | 7.7% (2) |  |
| Verbal Aggression | 3.8% (1) |  |
| Physical Aggression | 3.8% (1) |  |
| Suicidal Ideation | 3.8% (1) |  |
| Stress | 11.5% (3) |  |
| Non-Support | 3.8% (1) |  |
| Treatment Rejection | 0.0% (0) |  |
| **Scales Assessing Interpersonal Features** | |  |
| Dominance | 3.8% (1) |  |
| Warmth | 0.0% (0) |  |
